# Regional differences in astrocytic Aquaporin-4 protein levels and distribution in aging and Alzheimer’s disease in down syndrome

**DOI:** 10.1016/j.nbd.2025.107114

**Published:** 2025-09-21

**Authors:** Cherie A. Stringer, Haley K.S. Miyasato, Kevin A. Camey, Krina A. Ghadia, Elizabeth J. Andrews, Phong T. Ngo, Jesse R. Pascual, Sierra T. Wright, Justine A. Silva, Brianna M. Gawronski, Lourdes Gonzalez, Kevin A. Wood, Michael J. Phelan, Florence Lai, Frederick A. Schmitt, Jordan P. Harp, Adam M. Brickman, Patrick J. Lao, Mark E. Mapstone, Julia K. Kofler, Milos D. Ikonomovic, Elizabeth Head

**Affiliations:** a Department of Pathology and Laboratory Medicine, University of California, Irvine, CA, USA; b Alzheimer’s Disease Research Center, University of California, Irvine, CA, USA; c University of California, Irvine Institute for Memory Impairments and Neurological Disorders, CA, USA; d Alzheimer Biomarker Consortium-Down Syndrome, University of California, Irvine, CA, USA; e Center for Aging Research in Down Syndrome, University of California, Irvine, CA, USA; f Taub Institute for Research on Alzheimer’s Disease and the Aging Brain, Gertrude H. Sergievsky Center, and the Department of Neurology, Vagelos College of Physicians and Surgeons, Columbia University, New York, NY, USA; g Massachusetts General Hospital, Harvard Medical School, Boston, MA, USA; h Department of Neurology and Psychiatry, University of Pittsburgh School of Medicine, Pittsburgh, PA, USA; i Geriatric Research Education and Clinical Center, VA Pittsburgh Healthcare System, Pittsburgh, PA, USA; j University of Kentucky Department of Neurology, Lexington, KY, USA

**Keywords:** Astrocytes, beta-amyloid, Glia, Aging, Neurodegeneration, Trisomy 21

## Abstract

Aquaporin-4 (AQP4) is implicated in Alzheimer’s disease (AD) pathology through its role in astrocytic function, cerebrovascular integrity, and beta-amyloid (Aβ) clearance. Impaired Aβ clearance in AD is linked to changes in AQP4 distribution; however, the role of AQP4 in AD associated with Down Syndrome (DS) is poorly understood. This study investigates AQP4 protein levels, its relationship with Aβ deposition, and distribution patterns in DS. Using human post-mortem brain sections from the frontal and occipital cortex, we analyzed AQP4 and Aβ levels in samples from neurotypical controls, DS, DS with AD (DSAD), and late onset AD (LOAD). Protein levels and distribution were assessed using immunohistochemistry and immunofluorescence with quantitative imaging tools. AQP4 protein levels were higher with age in both neurotypical control and DS groups, but not in the LOAD group. AQP4 and Aβ were positively correlated with age in the frontal cortex of all groups. AQP4 and Aβ were positively correlated with each other after adjusting for age in the frontal cortex in both the control and DS groups which was not observed in the occipital cortex. In the frontal cortex of both DS and DSAD, AQP4 was more frequently distributed to the soma and proximal branches and less to astrocytic endfeet compared to the control group, consistent with previous reports of impaired glymphatic clearance and perivascular regulation. These findings support a relationship between altered AQP4 protein levels and distribution, Aβ accumulation, and region-specific vulnerability in DS and AD.

## 1. Introduction

Alzheimer’s disease (AD) is prevalent in individuals with Down syndrome (DS) (Fortea et al., 2021). The triplication of chromosome 21 (Trisomy 21) in DS is associated with the development of AD (Prasher et al., 1998; Doran et al., 2017). Trisomy 21 involves the overexpression of the amyloid precursor protein gene, which promotes excessive beta-amyloid (Aβ) production, resulting in the development of AD pathology by age 40 years (Videla et al., 2022). AD neuropathology and progression in DS are age-dependent, with clinical symptoms of dementia typically emerging after the age of 50 years (Videla et al., 2022; Alzheimer’s Disease International, 2024).

As of 2024, an estimated 55 million individuals worldwide, including many with DS, are affected by AD, with projections rising to 152 million by 2050 (Alzheimer’s Disease International, 2024). This increasing prevalence escalates healthcare costs and places significant social and financial burdens on families and caregivers, highlighting the urgent need for effective treatments. Two FDA-approved Aβ immunotherapies have recently become available, but individuals with DS have historically been excluded from AD (Cummings et al., 2024; U.S. Food and Drug Administration, 2024; Baumer et al., 2022; O’Neill, 2024; Strydom et al., 2018). The mechanisms underlying AD remain complex, and whether molecular pathways overlap between AD in the general population and DS-associated AD is still under investigation. These gaps emphasize the need for a deeper understanding of the cellular mechanisms involved in AD and DS, particularly a possible role for astrocytes, which have emerged as key players in AD pathology (Kim et al., 2024; Liu et al., 2025; Medscape, 2024).

Astrocytes are glial cells that support neuronal functions including synaptogenesis, neurotransmitter clearance to promote long-term potentiation, and the exchange of nutrients and waste, including Aβ, at the blood-brain barrier (BBB) via the glymphatic system (Verkhratsky et al., 2021; Díaz-Castro et al., 2023; Sofroniew and Vinters, 2010). In AD, Aβ plaque accumulation disrupts these crucial processes, contributing to neuroinflammation, BBB impairment, diminished synaptic functionality, and cognitive decline (Liddelow and Barres, 2017; Fagiani et al., 2021).

Aquaporin-4 (AQP4), a water channel predominantly expressed at the astrocytic endfeet along the BBB, has emerged as a contributor to AD progression (Jessen et al., 2015; Wilcock et al., 2009). Several studies in AD mouse models and human autopsy tissue highlight the link between Aβ plaque deposition and impaired cognition with altered AQP4 distribution from endfeet at the BBB (Wilcock et al., 2009; Smith et al., 2019; Zeppenfeld et al., 2017). In AD mouse models, loss of AQP4 reduces astrocyte–plaque association and results in more diffuse plaques with dystrophic neurites, underscoring the importance of astrocytic AQP4 in modulating Aβ pathology (Smith et al., 2019). Despite the established role of AQP4 in AD pathology in the neurotypical population, whether AQP4 contributes similarly to AD pathogenesis in DS remains unclear (Liddelow and Barres, 2017; Fagiani et al., 2021; Jessen et al., 2015).

Although AQP4 has been implicated in AD progression, its contribution to DS-associated AD remains poorly understood. To address this gap, we aimed to investigate AQP4 and Aβ expression and distribution across frontal and occipital cortices—regions affected early and late in AD, respectively (Thal et al., 2002; Grothe et al., 2017). To our knowledge, this is the first study to investigate AQP4 and Aβ protein levels across both frontal and occipital cortices in DS, addressing a key knowledge gap regarding region-specific astrocytic involvement in DS-associated AD.

We hypothesized that AQP4 and Aβ protein levels would increase with age and with AD pathology, that AQP4 and Aβ would be positively correlated, and that AQP4 would exhibit altered distribution from astrocytic endfeet in DS with AD (DSAD). To test these hypotheses, we evaluated astrocytic AQP4 protein levels and distribution in neurotypical controls, individuals with DS, individuals with DSAD, and individuals with late-onset AD (LOAD). Using immunohistochemistry, immunofluorescence, and QuPath, we assessed protein expression levels and distribution in human post-mortem brain samples, focusing on both the frontal and occipital cortices.

## 2. Materials and methods

### 2.1. Human post-mortem brain tissue

This study included frontal (*n* = 106), and occipital (*n* = 94) cortex post-mortem human brain tissue samples obtained from the Neuropathology Core of the Alzheimer’s Disease Research Center (ADRC) at the University of California, Irvine and the NIH NeuroBioBank. The frontal cortex was included as it is a site of early Aβ accumulation. The occipital cortex was included as it is affected late with AD pathogenesis and is also a region sensitive to cerebrovascular pathology. At the UCI ADRC, participants provided pre-mortem consent for the use of their brain tissue in research studies, obtained with Institutional Review Board approval. Tissue was initially fixed with 4 % paraformaldehyde at 4 ^◦^C for 14 to 24 days. Tissue obtained from the NIH NeuroBioBank was fixed in 10 % formalin, though the length of time in fixation was not available. Fixation durations in formalin and specific handling procedures may vary between individual brain banks within the NeuroBioBank network. Following fixation, all tissue was sectioned, then placed in 1× PBS with Sodium Azide (0.02 %) at 4 °C for long-term storage until use. Demographic data for the six diagnostic groups used in this study- young control (YC), middle-aged control (MC), aged control (AC), DS, DSAD, and LOAD- along with their sex distribution, age range, and post-mortem interval (PMI) for both cortical regions are listed in Table 1. Per available neuropathology reports, cases with additional primary neurodegenerative diagnoses or major focal lesions (e.g., large infarcts, primary brain tumors) were not included. Cause of death and systemic comorbidities were recorded when available but were not uniformly documented across donors and therefore were not used as covariates. Cases were selected as follows: YC and MC were from the NIH Neurobiobank, do not have clinical information or neuropathology assessments but were age and sex matched to DS and DSAD cases. DS cases were selected on the basis of age (<40 years) but do not have clinical nor neuropathology data available from the NIH Neurobiobank. AC cases were from the UCI ADRC and selected for a clinical diagnosis of not demented with insufficient neuropathology for AD. LOAD cases were from the UCI ADRC selected for the presence of both clinical dementia and AD neuropathology. DSAD cases were from the UCI ADRC to have a clinical diagnosis of dementia with AD neuropathology. Age matching with respect to tissue from individuals with DSAD and individuals with LOAD was not possible as individuals with DS do not live as long as those with LOAD (Esbensen, 2010). Therefore, the DSAD and LOAD samples were matched according to average PMI. Post-mortem brain tissue from the frontal and occipital cortices was obtained from the same individuals whenever available. However, due to limitations in tissue availability, not all cases had matched samples from both regions, leading to slight discrepancies in sample sizes across analyses (Table 1). Tissue selection criteria remained consistent across both regions to ensure comparability.

### 2.2. Immunolabeling

#### 2.2.1. Immunohistochemistry

Immunohistochemistry (IHC) was conducted using formalin-fixed post-mortem human brain tissue sections from occipital and frontal cortices. All tissue was sliced at 30 μm using a Leica Vibratome (Model# VT10005) and were stained using a free-floating method, as described here. Tissue was washed in 1× TBS (Bioland Scientific LLC, cat# TBS01–03) then, incubated in citrate buffer (0.0147 g/ml sodium citrate; Fisher Chemical, cat# S279–500) at a pH of 6 for 30 min at 80 °C in a water bath. Tissue was washed once in 1× TBS and then was incubated in 90 % Formic Acid (Spectrum Chemical MFG Corp, cas#F1091) for 10 min at RT. Tissue was washed three times in 1× TBS, 5 min per wash then incubated in 3 % Hydrogen Peroxide (Fisher Scientific, cat# H325–500) with 10 % Methanol (J.T. Baker, cat# 9093–03) in 1× TBS. Tissue was washed in 1× TBS and then in TBS-A (1× TBS with 0.1 % Triton-X100 (Fisher BioReagents, cat# BP151–100)) for 15 min at RT, followed by TBS-B (1× TBS with 5 % BSA (Gemini Bio cat# 700–100P) for 30 min at RT. Tissue was blocked in TBS-B with 5 % normal horse serum (Vector Laboratories, cat# S-2000–20) for 1 h and then incubated in primary antibody. Primary antibodies used in IHC include rabbit anti-AQP4 at a 1:10,000 dilution (ThermoFisher Scientific, cat# PA5–53234) and mouse anti-β-Amyloid (clone 6E10) at a 1:4000 dilution (Biolegend, cat# 803003) overnight at 4 °C on an orbital shaker. Tissue was washed in TBS-A for 5 min and then washed in TBS-B for 10 min. Tissue was incubated in secondary antibody (biotinylated horse anti-rabbit antibody (Vector Laboratories, cat# BA-1100) at a 1:1000 dilution or biotinylated horse anti-mouse (Vector Laboratories, cat# BA-2000) at a 1:1000 dilution) for 1 h at RT then, washed in 1× TBS for 5 min before incubation in ABC reagent (Vector Laboratories, Vectastain Elite ABC Kit, Peroxidase, Standard, cat#PK-6100) for 1 h at RT. Tissue was washed 3 times in 1× TBS for 5 min per wash then incubated in for 7 min at RT (Vector Laboratories, DAB Substrate Kit, Peroxidase (with Nickel), cat# SK-4100). Tissue was then mounted to FisherBrand SuperFrost Plus slides (Fisher Scientific, cat#12–550–15) and allowed to dry overnight. For counterstain, mounted tissue was incubated in Cresyl Violet Stain Solution, 1 % (Abcam, cat# AB246817) for 3 min, washed in DI water for 10 min, then dehydrated in 50 %, 70 %, 95 %, and 100 % Ethanol (Gold Shield, Ethyl Alcohol, Proof 200), for 10 min at each concentration. Mounted tissue was then incubated twice in Xylene (Xylenes Histological grade, Fisher Scientific, cat# X3P-1GAL) for 10 min each incubation. Mounted and counterstained tissue was cover slipped using DPX mounting media (Electron Microscopy Sciences, cat#13512) and Fisherbrand Premium Cover Glasses coverslips (Fisher Scientific, cat# 12–548-5 J). Slides were allowed to dry overnight before whole slide imaging as described in the imaging segment of the methods section. Negative controls omitting the primary antibody were included in each staining batch and consistently showed no detectable signal.

#### 2.2.2. Immunofluorescence

Immunofluorescence (IF) was conducted using formalin-fixed post-mortem human brain tissue sections from occipital and frontal cortices. All tissue was sliced at 30 μm thick using the Leica Vibratome (Model# VT10005) and were stained using the following free-floating method, as described here. Tissue was washed in 1× TBS (Bioland Scientific LLC, cat# TBS01–03) for 5 min at RT twice. Tissue was then incubated in citrate buffer (0.0147 g/ml sodium citrate; pH 6.0; Fisher Chemical, cat# S279–500) for 30 min in a water bath at 80 °C then allowed to cool for 10 min at RT. Tissue was washed once in 1× TBS and then was incubated in 90 % Formic Acid (Spectrum Chemical MFG Corp, cas#F1091) for 10 min at RT. Tissue was washed in 1× TBS twice at RT for 5 min per wash, washed in TBS-A (1× TBS with 0.1 % Triton-X100 (Fisher Scientific, cat# BP151–100)) for 15 min, then incubated in TBS-B (1× TBS with 5 % BSA (Gemini Bio, cat# 700-100P) for 30 min at RT. Tissue was blocked in TBS-B with 5 % normal horse serum (Vector Laboratories, cat# S-2000-20) for 1 h at RT and then incubated in primary antibodies overnight at 4 °C on an orbital shaker. Primary antibodies utilized included, Chicken anti-Aβ IgY (Origene, cat# AP31802PU-N) at a 1:500 dilution, Mouse anti-AQP4 (LS-Bio, LS-C413484–100) at a 1:200 dilution, Sheep anti-CD31/PECAM1 (R&D Systems, cat# AF806) at a 1:200 dilution, and Rabbit anti-Collagen IV (Abcam, cat# ab214417). Secondary antibodies used included, Donkey anti-Chicken IgY Alexa fluor 488 (Thermofisher Scientific, cat# A78048), Donkey anti-Sheep 555 (Fisher Scientific, cat# A21436), Donkey anti-Rabbit 594 (Fisher Scientific, cat# A21207) Donkey anti-Mouse Alexa fluor 647 (Fisher Scientific, cat# A-31751). Tissue was washed twice in TBS-A for 5 min per wash and then washed in TBS-B for 15 min. All secondaries were diluted 1:1000 in blocking buffer (1× TBS-B with 5 % normal horse serum). Tissue was washed in 1× TBS for 5 min then incubated in 1× True Black Lipofuscin Autofluorescence Quencher (diluted in 70 % ethanol; Biotium, cat# 23007) for 4 min. Tissue was washed in 1× TBS then mounted to FisherBrand SuperFrost Plus slides (Fisher Scientific, cat# 12–550-15). Slides were coated with Fluoromount mounting medium with DAPI (Vector Laboratories, VECTA-SHIELD Vibrance antifade mounting medium, cat# H-1800). Negative controls omitting the primary antibody were included in each staining batch and consistently showed no detectable signal. Immunofluorescence analyses for AQP4 distribution across AC, DS, DSAD, and LOAD were performed using a high-powered microscope detailed in the imaging portion of the Methods section.

### 2.3. Imaging

#### 2.3.1. Confocal microscopy

Slides were imaged using a Zeiss LSM 900 with Airyscan confocal microscope at 40× magnification, capturing *Z*-stack images with 1 μm step intervals across a total depth of 10 μm within 30 μm thick tissue slices to minimize background. The Airyscan processing feature was applied to each 1 μm slice to enhance resolution. Prior to imaging, detector gain (PMT) and laser power were optimized for each channel to avoid saturation and then held constant within each imaging batch. Channels were acquired with the following excitation/emission (detector) windows: DAPI: Ex 405 nm, Em 420–480 nm (gain 850 V); Alexa Fluor 488/GFP: Ex 488 nm, Em 500–550 nm (gain 850 V); TRITC/Alexa Fluor 555: Ex 561 nm, Em 565–605 nm (gain 850 V); Texas Red/Alexa Fluor 594: Ex 594 nm, Em 600–650 nm (850 V); Cy5/Alexa Fluor 647: Ex 640 nm, Em 660–720 nm (gain 850 V). Emisssion bandpasses were chosen to minimize spectral overlap and improve signal specificity; signle-labeled controls and sequential scanning were used to verigy negligible bleed-through. Post-imaging, white light laser exposure was fine-tuned for each channel in the frontal and occipital cortices, with specific settings for each channel. Finally, the orthogonal array feature was applied to compress the Z-stack images.

#### 2.3.2. Whole slide imaging

We acquired whole slide images of human post-mortem brain tissue by scanning the entire mounted tissue slice following IHC using the Aperio Versa 200 digital slide scanner (Leica Biosystems 23VER200BFC001) at up to 40× objective in brightfield. These high-resolution digital images were then used for detailed quantification via QuPath.

#### 2.4. QuPath quantification

QuPath (v0.3.0) was used to generate a pixel classifier for quantifying AQP4 expression from whole-slide IHC images. The classifier was trained on ten representative AQP4-labeled images, encompassing a range of staining intensities across all diagnostic groups. Training involved color deconvolution and annotation of representative regions of interest (ROIs) across cortical gray matter using QuPath’s pixel classifier tool. Annotations were explicitly labeled as either positive (AQP4+; astrocyte-rich areas with clear AQP4 staining) or negative (background or unstained tissue). A Random Trees (RTrees) classifier was used to distinguish positive from negative pixels. Training was performed at moderate resolution with feature types including Gaussian, weighted deviation, gradient magnitude, and Hessian determinant at scales of 0.5 and 1.0. Local normalization was disabled, and live prediction was used to confirm classifier performance prior to quantification. Classifier performance was validated by visually confirming output on excluded test images. Annotation regions and pixel-classification overlays are shown in Supplement Fig. 1.

For quantification, whole-slide images were imported into QuPath and five 600 μm^2^ boxes were manually placed across gray matter regions per case, avoiding white matter and artifacts. The trained classifier was then applied to each annotated box to calculate the percentage of AQP4-positive pixels. Percent positivity for each case was calculated by summing all AQP4-positive pixels across the five boxes, dividing by the total pixel count across all boxes, and multiplying by 100. The final values were exported and analyzed in GraphPad Prism v9.0.

The AQP4 pixel classifier, along with full usage instructions, is publicly available at: https://github.com/StringerCA/Stringer-AQP4-Classifier-QuPath

### 2.5. ImageJ Validation of QuPath Quantification

To validate the QuPath-derived AQP4 quantification, an independent pixel-based analysis was performed using ImageJ (v1.54). For each diagnostic group, cases representing the highest and lowest AQP4 expression based on QuPath classifier output were selected to ensure both high- and low-expression ROIs were analyzed. Representative AQP4 IHC-stained ROIs from each case (600 μm^2^, matched to QuPath ROIs) were imported and processed using color deconvolution (H-DAB). For each ROI, a fixed intensity threshold was applied to isolate DAB signal, using one of two pre-established threshold ranges depending on overall signal intensity (low-expression: min = 120, max = 175; high-expression: min = 0, max = 30). These thresholds were determined empirically using reference images across diagnostic groups. The % positive area was recorded directly from the ImageJ threshold output for each ROI and compared to the corresponding QuPath-derived % positive pixel value. No additional area calculations were performed. ImageJ values were compared to QuPath classifier-derived % positive pixel values using Pearson correlation and simple linear regression in GraphPad Prism v9.0. A representative ROI with corresponding QuPath and ImageJ segmentation overlays is shown in Supplement Fig. 2 A. Correlation plots for frontal and occipital cortex comparisons are shown in Supplement Fig. 2B.

### 2.6. Semi-quantitative analysis of AQP4 distribution

All IHC sections for both frontal and occipital regions were analyzed semi-quantitatively for AQP4 distribution to determine the percentage of ‘Endfoot’- and ‘Non-Endfoot’- associated (NEF) distributions across all diagnostic groups (i.e., aged control, DS, DSAD, and LOAD) by two blinded observers. Additionally, all cases in which there was no immunostaining for AQP4 was categorized as ‘No AQP4.’ Blinded observers were blinded as to the cases and diagnostic groups. Blinded observers examined whole slide images corresponding to all cases and recorded the most predominant AQP4 distribution observed within each of the cortical layers I, II, III, V, VI, and the gray matter/white matter junction. These distributions across layers were averaged to determine the overall predominant distribution of AQP4 in each tissue sample across diagnostic groups. Distribution was reported as the mean percentage for each diagnostic group (Fig. 3). Diagnostic and case demographics are detailed in Supplement Tables 1 and 2. Although we examined AQP4 distribution across individual cortical layers, no consistent layer-specific patterns emerged across diagnostic groups. Therefore, we summarized data at the case level, which better represents overall group differences.

### 2.7. 3D rendering

Confocal *Z*-stack images with a 10 μm z-step interval were processed using IMARIS 10.1.0 software. For 3D rendering, the ‘Surface Rendering’ feature was applied to each channel individually. The DAPI channel, for example, was rendered by selecting the ‘Surface’ feature and adjusting the surface representation incrementally to ensure accuracy. The channel color was set to blue, and the final rendering was saved. This procedure was consistently applied across all channels to maintain uniformity in 3D rendering across diagnostic groups.

### 2.8. Statistical analyses

ANOVA, Mann-Whitney *U* tests, non-linear and linear regression, Pearson correlation and Spearman’s rho, and residual analyses were used to test our hypotheses, with corresponding graphs generated using GraphPad Prism software (version 10). For direct comparisons of AQP4 and Aβ protein levels, values were natural log-transformed for optimal visualization and analyzed using Pearson correlation. Differences in AQP4 and Aβ protein levels across the six diagnostic groups were assessed using one-way ANOVA followed by Tukey’s post-hoc test for multiple comparisons. Residual analyses were performed to evaluate the association between AQP4 and Aβ independent of age. Residuals for AQP4 and Aβ protein levels adjusting for age were computed via linear regression and Pearson correlation. Sex differences in AQP4 protein levels were analyzed using the Mann-Whitney *U* test due to non-normally distributed data. Non-linear regression and Spearman’s rho were used to assess relationships between AQP4 and Aβ protein levels with PMI and age. Statistical significance was set at *P* ≤ 0.05. A priori, we planned to compare DSAD and LOAD across all outcome measures.

## 3. Results

AQP4 protein levels were assessed across six demographic and histopathological groups in both the frontal and occipital cortices. The frontal cortex samples included 17 YC, 19 MC, 18 AC, 16 DS, 17 DSAD, and 19 LOAD cases. Occipital cortex samples included 18 YC, 18 MC, 16 AC, 11 DS, 17 DSAD, and 14 LOAD cases.

We first evaluated the association of PMI with AQP4 and Aβ protein levels by including all cases in the analysis (Supplement Fig. 3). Negative correlations were observed between both AQP4 and Aβ with post-mortem interval (PMI) across all diagnostic groups in the frontal (% AQP4: *r* = − 0.44, *p* < 0.001; %Aβ: *r* = − 0.33, *p* = 0.001) and occipital cortex (%AQP4: *r* = − 0.35, *p* = 0.007; %Aβ: *r* = − 0.32, *p* = 0.015), indicating that PMI did not meaningfully impact AQP4 or Aβ protein levels following non-linear regression and Spearman’s rho analyses (Supplement Fig. 3).

The association of sex with AQP4 levels was examined within the neurotypical control cases and separately within the DS cases (Supplement Fig. 4). No sex differences in AQP4 protein levels were detected across any group, except in the occipital cortex of the neurotypical control group, where AQP4 protein levels were higher in females than in males (Mann-Whitney U test, *U* = 237.5, *p* = 0.017) (Supplement Fig. 4).

### 3.1. AQP4 and Aβ protein levels are higher with age and at earlier ages in DS

**Fig. 1.**
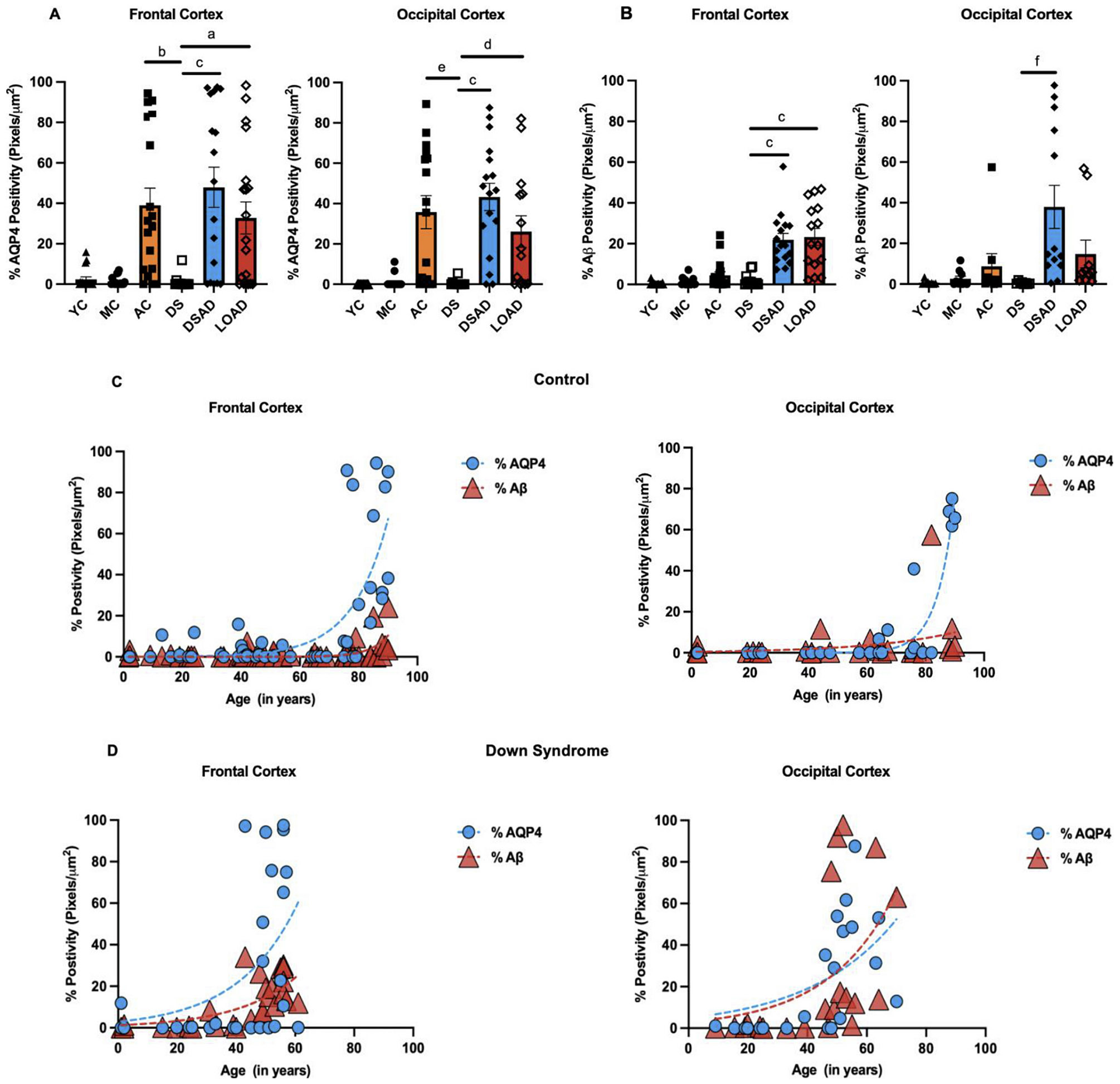
AQP4 and Aβ Protein Levels are Higher in DSAD Than in DS and is Correlated with Age. (A) AQP4 protein levels were higher in DSAD compared to DS, YC, and MC (frontal cortex: *F*(5, 94) = 11.48, *R*^*2*^ = 0.40, *p* < 0.001; occipital cortex: *F*(5, 84) = 13.48, *R*^*2*^ = 0.44, *p* < 0.001). However, AQP4 levels did not differ between DSAD and LOAD (frontal cortex: *p* = 0.38; occipital cortex: *p* = 0.51) or between DSAD and AC (frontal cortex: *p* = 0.92; occipital cortex: *p* = 0.91). (B) Aβ protein levels were higher in DSAD than in DS, YC, MC, and AC (frontal cortex: *F*(5, 92) = 23.95, *R*^*2*^ = 0.60, *p* < 0.001; occipital cortex: *F*(5, 52) = 4.989, *R*^*2*^ = 0.324, *p* = 0.005). However, Aβ levels in DSAD were comparable to LOAD (frontal cortex: *p* = 0.99; occipital cortex: *p* = 0.13). (C) AQP4 and Aβ protein levels were correlated with age in the control group in the frontal cortex (AQP4: *n* = 53, *r* = 0.60, *R*^*2*^ = 0.536, *p* < 0.001; Aβ: n = 53, *r* = 0.30, *R*^*2*^ = 0.30, *p* = 0.03) but only AQP4 protein levels were correlated with age in the occipital cortex (AQP4: *n* = 50, *r* = 0.68, *R*^*2*^ = 0.86, *p* < 0.001; Aβ: *n* = 24, *r* = 0.34, *R*^*2*^ = 0.08, *p* = 0.082). (D) Both AQP4 and Aβ were positively correlated with age in the DS group in the frontal cortex (AQP4: *n* = 30, *r* = 0.58, *R*^*2*^ = 0.26, *p* = 0.001; Aβ: n = 30, *r* = 0.76, *R*^*2*^ = 0.50, *p* < 0.001) and occipital cortex (AQP4: *n* = 28, *r* = 0.70, *R*^*2*^ = 0.31, *p* < 0.001; Aβ: *n* = 22, *r* = 0.50, *R*^*2*^ = 0.27, *p* < 0.001). (A, B) Group comparisons were performed using one-way ANOVA with Tukey’s post-hoc test (significance markers: ^a^ *p* = 0.011; ^b^ *p* = 0.001; ^c^ *p* < 0.001; ^d^ *p* = 0.04; ^e^ *p* = 0.001; ^f^ *p* = 0.005). (C, D) Correlations were analyzed using non-linear regression and Spearman’s rho. All raw data are available upon request.

To test the hypothesis that AQP4 protein levels increase with age and AD pathology in DS, we performed immunohistochemistry on post-mortem brain tissue from frontal and occipital cortices and quantified AQP4 and Aβ levels using QuPath (Supplement Fig. 1) (Bankhead et al., 2017; Cell Classification, 2024; Pixel Classification, 2024a; Pixel classification, 2024b). One-way ANOVA and Tukey’s post-hoc analysis was initially performed to assess unadjusted group differences (Fig. 1A, B). We found that AQP4 protein levels were highest in AC, DSAD, and DS in both the frontal (*F*(5, 94) = 11.48, *R^2^* = 0.40, *p* < 0.001) and occipital cortex (*F*(5, 84) = 13.48, *R^2^* = 0.44, *p* < 0.001) (Fig. 1A). Additionally, we found that Aβ was significantly higher in DSAD and LOAD than in DS in the frontal cortex (*F*(5, 92) = 23.95, *R^2^* = 0.60, *p* < 0.001) and higher in DSAD than DS in the occipital cortex (*F*(5, 52) = 4.989, *R^2^* = 0.324, *p* < 0.001). These findings suggest that AQP4 levels are elevated in aged controls and disease-associated groups.

Since AQP4 protein levels were higher in AC, DSAD, and LOAD classes, we wanted to determine if AQP4 and Aβ levels increase with age. Non-linear regression and Spearman’s rho analyses confirmed that AQP4 and Aβ levels were higher with age, with a notable rise after 70 years in the control group frontal cortex (%AQP4: *r* = 0.60, *R^2^* = 0.536, *p* < 0.001; %Aβ: *r* = 0.30, *R^2^* = 0.30, *p* = 0.03) (Fig. 1C). AQP4 was also positively associated with age in the occipital cortex (%AQP4: *r* = 0.68, *R^2^* = 0.86, *p* < 0.001) but Aβ was not (%Aβ: *r* = 0.34, *R^2^* = 0.08, *p* = 0.082) (Fig. 1C). In the DS group, both proteins were positively associated with age in frontal cortex (%AQP4: *r* = 0.58, *R^2^* = 0.26, *p* = 0.001; %Aβ: *r* = 0.76, *R^2^* = 0.50, *p* < 0.001) and occipital cortex (%AQP4: *r* = 0.70, *R^2^* = 0.31, *p* < 0.001; Aβ: *r* = 0.50, *R^2^* = 0.27, *p* < 0.001), with an exponential rise after 45 years (Fig. 1D).

### 3.2. AQP4 Protein Levels are Correlated with Aβ in a Brain Region-Specific Manner

**Fig. 2.**
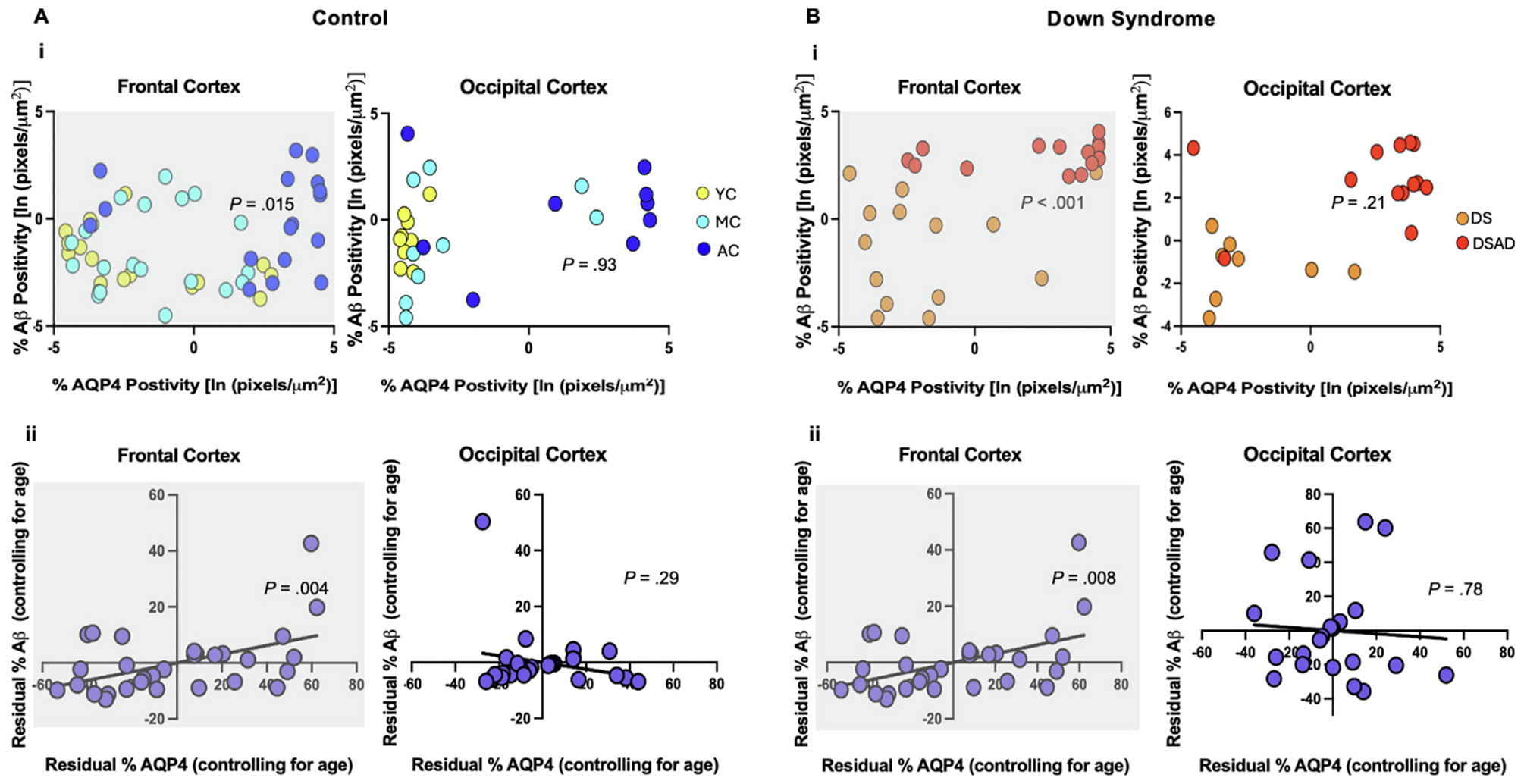
AQP4 and Aβ Protein Levels are Correlated in a Brain Region-Specific Manner. (Ai) In the control group, AQP4 protein levels were positively correlated with Aβ in the frontal cortex (n = 53, *r* = 0.33, *R*^*2*^ = 0.111, 95 % CI 0.07 to 0.55, *p* = 0.015) but not in the occipital cortex (*n* = 27, *r* = − 0.02, *R*^*2*^ = 0.000, 95 % CI − 0.39 to 0.37, *p* = 0.93). (Bi) In the Down syndrome group, AQP4 protein levels were positively correlated with Aβ in the frontal cortex (n = 30, *r* = 0.62, *R*^*2*^ = 0.385, 95 % CI 0.34 to 0.80, *p* < 0.001) but not in the occipital cortex (*n* = 21, *r* = 0.28, *R*^*2*^ = 0.08, 95 % CI −. 017 to 0.64, *p* = 0.21). (Aii) After controlling for age, AQP4 remained significantly correlated with Aβ in the frontal cortex of the control group (n = 53, *r* = 0.39, *R*^*2*^ = 0.148, 95 % CI 0.13 to 0.59, *p* = 0.004) but not in the occipital cortex (n = 27, *r* = − 0.22, *R*^*2*^ = 0.047, 95 % CI − 0.55 to 0.18, *p* = 0.29). (Bii) Similarly, in the Down syndrome group, AQP4 remained significantly correlated with Aβ in the frontal cortex after controlling for age (n = 30, *r* = 0.48, *R*^*2*^ = 0.226, 95 % CI 0.14 to 0.71, *p* = 0.008) but not in the occipital cortex (n = 21, *r* = − 0.07, *R*^*2*^ = 0.004, 95 % CI − 0.48 to 0.38, *p* = 0.78). (Ai-Bi) Data were natural log-transformed (ln) for visualization, with axes labeled in logarithmic terms (ln) rather than raw percentages. Axes are scaled independently to best represent the range of values within each region. Analyses were performed using Pearson correlation. (Aii-Bii) Residual data were analyzed using linear regression and Pearson correlation to control for age.

To examine the relationship between AQP4 and Aβ protein levels, we assessed correlations between AQP4 positivity and Aβ positivity across control and DS groups in the frontal and occipital cortices via linear regression and Pearson correlation (Fig. 2). Positive correlations were observed between AQP4 and Aβ in the frontal cortex for both the control group (YC + MC + AC: *n* = 53, *r* = 0.33, *R^2^* = 0.111, 95 % CI 0.07 to 0.55, *p* = 0.015) and the DS group (DS + DSAD: *n* = 30, *r* = 0.62, *R^2^* = 0.385, 95 % CI 0.34 to 0.80, *p* < 0.001) only (Fig. 2 Ai-Bi). When controlling for age, significant positive correlations remained between AQP4 and Aβ in the frontal cortex for both the control (*r* = 0.39, *R^2^* = 0.148, 95 % CI 0.13 to 0.59, *p* = 0.004) and DS groups (*r* = 0.48, *R^2^* = 0.226, 95 % CI 0.14 to 0.71, *p* = 0.008), but no significant correlations were observed in the occipital cortex for either group (control: *r* = − 0.22, *R^2^* = 0.047, 95 % CI −0.55 to 0.18, *p* = 0.29; DS: *r* = − 0.07, *R^2^* = 0.004, 95 % CI − 0.48 to 0.38, *p* = 0.78) following analysis via linear regression and Pearson correlation (Figure 2Aii-Bii).

### 3.3. AQP4 is Distributed Away from Astrocytic Endfeet in DSAD

**Fig. 3.**
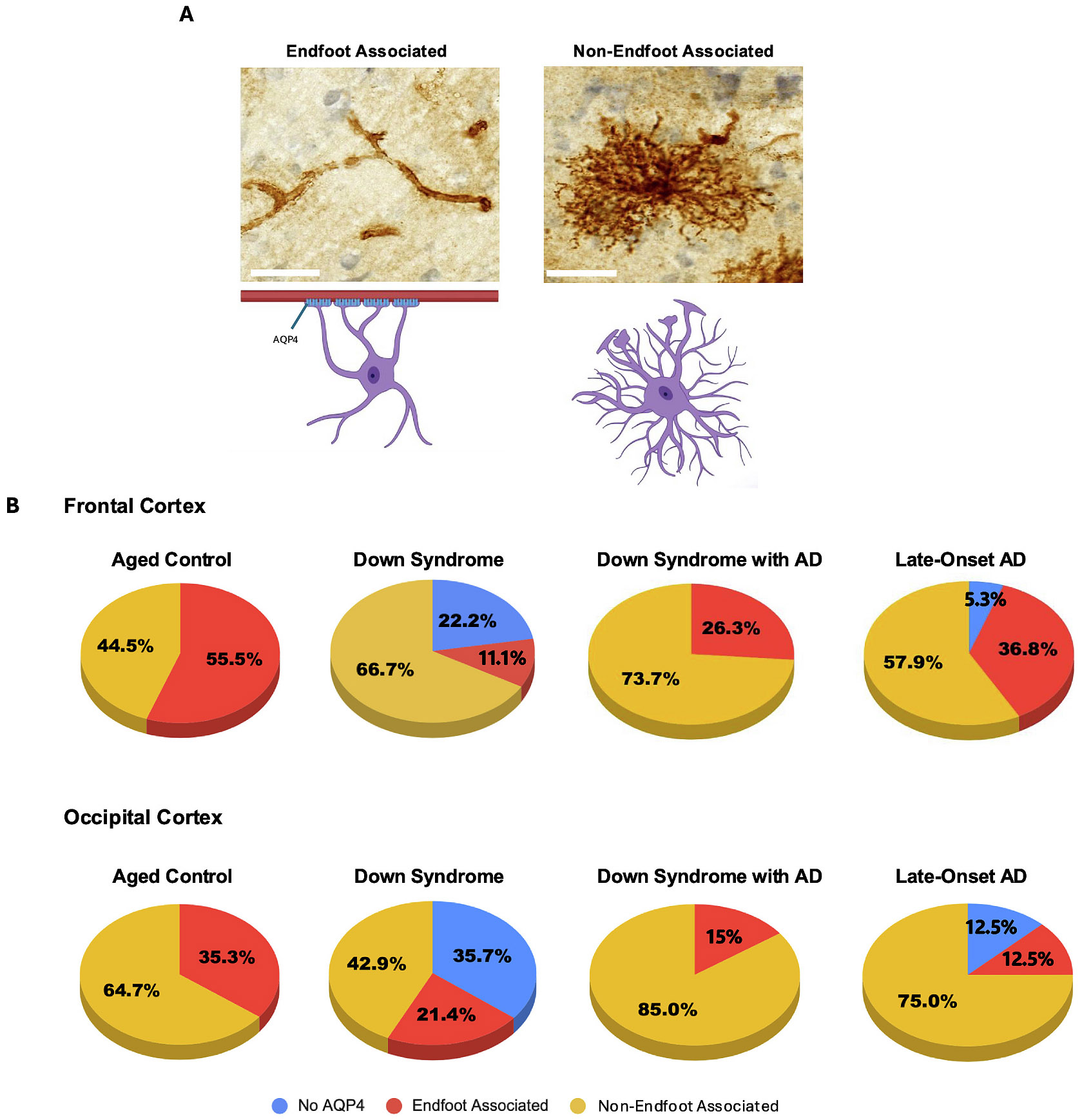
AQP4 is predominantly NEF-associated with AD pathology in DS. Immunohistochemistry of frontal and occipital cortex tissue from AC, DS, DSAD, and LOAD groups revealed three distinct AQP4 distribution patterns: ‘No AQP4,’ ‘Endfoot Associated,’ and ‘NEF Associated.’ Semi-quantitative analysis showed that AQP4 was primarily ‘NEF Associated’ in DS, DSAD, and LOAD groups, while it was predominantly ‘Endfoot Associated’ in AC in the frontal cortex. In the occipital cortex, AQP4 was mainly ‘NEF Associated’ in AC, DSAD, and LOAD groups, whereas in DS, AQP4 distribution was more evenly distributed between ‘Endfoot Associated’ and ‘NEF Associated.’ IHC images captured at 40× magnification; scale bars at 50 μm.

To assess whether AD pathology might influence the distribution of AQP4 away from astrocytic endfeet, we conducted immunohistochemistry and semi-quantitative analysis of AQP4 distribution in the frontal and occipital regions of a subset of AC, DS, DSAD, and LOAD groups (Fig. 3; Supplement Tables 1 and 2). AQP4 distribution was classified into three categories: ‘No AQP4,’ ‘Endfoot Associated,’ and ‘NEF Associated.’ ‘NEF Associated’ indicates AQP4 distribution across the soma and proximal branches and away from the astrocytic endfeet (Fig. 3A). ‘Endfoot Associated’ refers to patterns consistent with perivascular astrocytic endfeet, as indicated by alignment along structures resembling vasculature. Although vascular markers were not included in this study, these patterns are consistent with previously established AQP4 distribution in perivascular astrocytic endfeet (Kim et al., 2024; Jessen et al., 2015).

In the frontal cortex, a balanced distribution between ‘Endfoot Associated’ (55.5 %) and ‘NEF Associated’ (44.5 %) AQP4 distribution was observed in the AC group (Fig. 3B). In the DS group, ‘NEF Associated’ distribution was predominant (66.7 %), followed by ‘No AQP4’ (22.2 %) and a smaller fraction of ‘Endfoot Associated’ AQP4 (11.1 %). The highest occurrence of ‘NEF Associated’ distribution was observed in the DSAD group (73.7 %), with a notable proportion of ‘Endfoot Associated’ AQP4 also present (26.3 %). Similarly, in the LOAD group, ‘NEF Associated’ distribution was most frequent (57.9 %), along with a higher proportion of ‘Endfoot Associated’ AQP4 (36.8 %) compared to the DS group. These findings indicate increases in both ‘NEF Associated’ and ‘Endfoot Associated’ AQP4 distribution in DSAD and LOAD, potentially reflecting homeostatic and reactive astrocytic states.

In the occipital cortex, ‘NEF Associated’ distribution was predominant in the AC, DSAD, and LOAD groups (64.7 %, 85.0 %, and 75.0 %, respectively). In the DS group, ‘No AQP4’ (35.7 %) and ‘NEF Associated’ distribution (42.9 %) were most common, with the higher proportion of ‘No AQP4’ reflecting slightly lower overall AQP4 levels compared to the frontal cortex. Additionally, a higher proportion of ‘Endfoot Associated’ AQP4 was observed in the occipital cortex (21.4 %) in the DS group compared to the frontal cortex (11.1 %). These findings demonstrate distinct AQP4 distribution patterns across brain regions and diagnostic groups.

### 3.4. Immunofluorescence Illustrates AQP4 Distribution Differences Across Groups

**Fig. 4.**
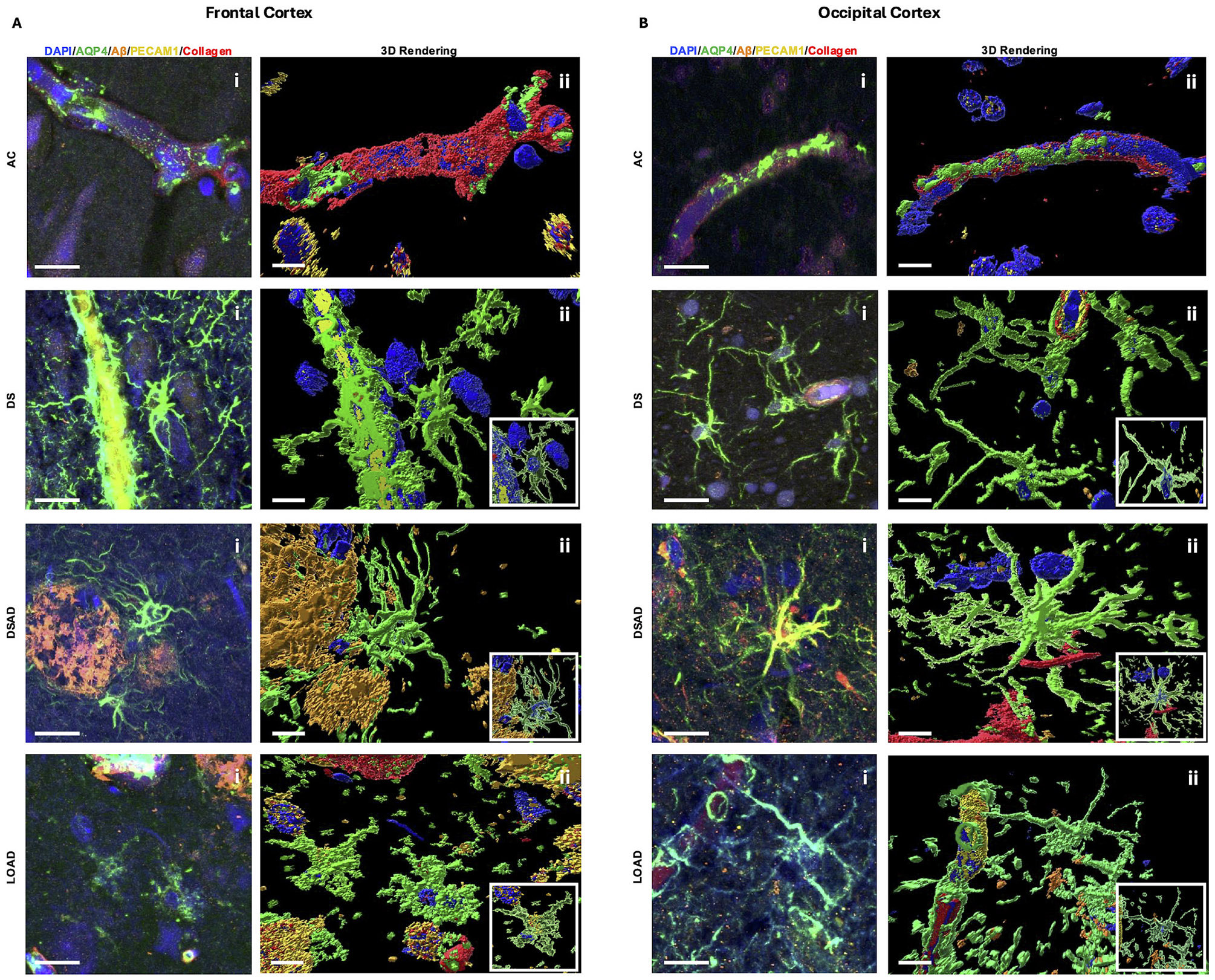
AQP4 is distributed to astrocytic soma and proximal branches in DS, DSAD, and LOAD groups. Immunofluorescence staining was performed for AQP4 (green), Aβ (orange), CD31/PECAM1 (yellow), Collagen IV (red), and DAPI (blue) in representative AC, DS, DSAD, and LOAD tissue slices from the frontal (A) and occipital (B) cortices. Tissue was imaged using confocal microscopy (i) and further enhanced with IMARIS 10.1.0 3D surface rendering to better visualize AQP4 distribution relative to vasculature and nuclei (ii). Inset images show magnified 3D renderings with AQP4 made transparent to highlight astrocyte nuclei colocalized with NEF-associated AQP4 (A, B, i–ii). In AC, AQP4 was primarily distributed to vascular endfeet in both cortices in the examples shown. In DS tissue, both endfoot-associated and NEF-associated patterns were represented. In DSAD and LOAD tissues, AQP4 was predominantly distributed across the astrocyte NEF, consistent with semi-quantitative analysis. Images captured at 40× magnification. Scale bars: (i) 10 μm and (ii) 7 μm.

To visualize and validate our semi-quantitative analysis of AQP4 distribution, we performed immunofluorescence for AQP4 and Aβ in tissue from both frontal and occipital regions across AC, DS, DSAD, and LOAD groups. Vascular markers PECAM1 and Collagen IV were included to confirm the perivascular localization of AQP4. Cases were selected to qualitatively represent predominant distribution patterns, including astrocytic endfoot- or NEF-Associated AQP4 distribution, and were used to visually support the IHC-based semi-quantitative analysis shown in Fig. 3 (Fig. 4).

To best illustrate AQP4 distribution, 3D-rendered images were generated and enlarged for enhanced visualization (Fig. 4A-Bii). In the AC group, the representative images highlight endfoot associated distribution; however, the semi-quantitative analysis in Fig. 3 indicates that NEFAssociated distribution was also common, particularly in the occipital cortex. In the DS group, AQP4 predominantly exhibited NEFAssociated distribution (Fig. 4A-B). Similarly, AQP4 distribution in tissues from the DSAD and LOAD groups was primarily NEFAssociated, consistent with semi-quantitative analysis (Fig. 4A-B).

## 4. Discussion

### 4.1. Summary of findings

We hypothesized that AQP4 and Aβ protein levels would increase with age and AD, that AQP4 and Aβ would be positively correlated, and that AQP4 would be distributed away from astrocytic endfeet in DSAD. This study provides new insights into the levels, distribution, and potential roles of AQP4 in the context of DS and AD. Age- and group-related differences were observed in both the frontal and occipital cortices. While the frontal cortex exhibited the most pronounced differences in the relationship between AQP4 and Aβ protein levels, distinct and meaningful differences were also observed in the occipital cortex, a region typically affected later in AD progression in both plaque and tangle pathology (Thal et al., 2002; Grothe et al., 2017). Although we note that the occipital cortex is affected earlier in disease by vascular pathology (Lao et al., 2024). These findings underscore the importance of examining both anterior and posterior regions to obtain a comprehensive understanding of AQP4 across age and AD pathology. Finally, with increasing age and AD pathology, there were higher levels of AQP4 across the soma and proximal branches and lower levels in astrocytic endfeet, reflecting variations in astrocytic distribution patterns and implicating changes to astrocytic states.

### 4.2. Regional- and Age-Associated Patterns of AQP4 and Amyloid Pathology

In the frontal cortex, both AQP4 and Aβ protein levels were higher with age in control and DS groups. However, in the occipital cortex, an age-related increase in AQP4 protein levels was observed in the control group, while Aβ levels did not differ by age. In contrast, both AQP4 and Aβ protein levels were higher with age in the DS group in this region. This regional difference may reflect the temporal progression of amyloid pathology, where the frontal cortex exhibits earlier and more advanced Aβ accumulation, consistent with Thal staging (Thal et al., 2002). Conversely, the occipital cortex typically accumulates Aβ at later stages in AD and at later ages in DS (Thal et al., 2002). These contrasting temporal profiles provided a rationale for selecting these regions, allowing us to examine both early- and late-vulnerable cortical areas.

The distinct temporal and regional patterns of Aβ pathology and AQP4 distribution in controls and LOAD may explain the varying correlations observed between AQP4 and Aβ across brain regions (Thal et al., 2002; Grothe et al., 2017; Lao et al., 2024). While previous studies reported a relationship between AQP4 and Aβ in the frontal cortex, this study is the first to demonstrate this correlation in both frontal and occipital cortices of individuals with DS. These findings highlight the importance of examining AQP4 dynamics in regions with delayed amyloid deposition, providing insights into potential mechanisms underlying glymphatic dysfunction in DS.

Recent neuroimaging studies of cerebrovascular pathology in DS suggest that enlarged perivascular spaces (PVS) may appear as early as 30 years of age, preceding amyloid deposition detectable by PET (Lao et al., 2024). Early PVS widening could reflect glymphatic dysfunction, with AQP4 potentially serving as a mechanistic link between PVS widening and Aβ accumulation.

These findings suggest a biphasic role for AQP4, where its initial upregulation at astrocytic endfeet supports Aβ clearance, while subsequent distribution to the soma and proximal branches observed in DSAD and LOAD may impair glymphatic function and contribute to Aβ accumulation. This conceptualization aligns with previous studies that suggest that AQP4 facilitates Aβ clearance and modulates amyloid deposition in a region-specific manner (Smith et al., 2019; Zeppenfeld et al., 2017; Iliff et al., 2012).

Age is a critical factor in the development of AD pathology in individuals with DS (Fortea et al., 2021; Esbensen, 2010). Trisomy 21 accelerates aging-related biological processes, including Aβ accumulation, with AD pathology present by age 40 and clinical symptoms typically appearing after the age of 50 (Lao et al., 2024; Wiseman et al., 2015). Our findings align with this trajectory, demonstrating that both AQP4 and Aβ protein levels increase earlier in DS (~45 years) compared to neurotypical controls (~75 years). Early onset of AQP4 upregulation underscores its potential role in the biological processes associated with aging that may contribute to heightened vulnerability to AD in DS. These data add to the growing body of evidence that age in DS strongly predicts AD development and progression (Silva et al., 2021).

### 4.3. Importance of AQP4 and Aβ Correlations in the Context of Regional and Pathological Dynamics

Positive correlations between AQP4 and Aβ protein levels were observed in the frontal cortex of the control and DS groups but not in the occipital cortex. This regional specificity aligns with established patterns of Aβ deposition, which typically begins in anterior regions like the frontal cortex and progresses to posterior regions, such as the occipital cortex, in later stages of AD (Thal et al., 2002; Grothe et al., 2017). These findings suggest that astrocytic responses to Aβ accumulation may vary with disease state and progression.

Before controlling for age and PMI, older control and DSAD cases clustered in the upper-right quadrant of scatter plots for both regions, indicating a stronger relationship between %AQP4 and %Aβ with advancing age and pathology. After adjustments, correlations persisted in the frontal cortex of control and DS groups, suggesting that the relationship is influenced by factors beyond aging alone, such as neurodegeneration-associated neuroinflammation. While intriguing, these correlations should be interpreted cautiously, as they do not establish causality. AQP4 dysfunction could plausibly contribute to perivascular and glymphatic changes observed in DS and AD; however, our findings remain correlational, and we did not directly assess glymphatic clearance in this study.

Nonetheless, animal studies provide mechanistic support for this link. The absence of AQP4 disrupts glymphatic clearance, resulting in diffuse amyloid plaque morphology, increased neuronal Aβ uptake, and heightened neuronal death (Fagiani et al., 2021; Smith et al., 2019; Zeppenfeld et al., 2017; Iliff et al., 2014). Additional mechanistic evidence from *Snta1−/−* and related mouse models further supports this framework, as loss of perivascular AQP4 anchoring impairs glymphatic transport and accelerates Aβ plaque formation (Mestre et al., 2018; Simon et al., 2022; Pedersen et al., 2023). While these models underscore the role of AQP4 in glymphatic function, the correlations observed in human data highlight the importance of other factors, such as neuroinflammation, in modulating the relationship between AQP4 and Aβ. Understanding these dynamics is critical to elucidate the role of AQP4 in DS and AD pathogenesis.

### 4.4. Astrocytic AQP4 Distribution and Its Implications for Amyloid Clearance and Neurodegeneration in DS

Our semi-quantitative analysis revealed distinct AQP4 distribution patterns across diagnostic groups, varying notably between the frontal and occipital regions. In the frontal cortex, the AC group predominantly exhibited ‘Endfoot Associated’ AQP4, consistent with preserved astrocytic function and efficient glymphatic clearance (Zeppenfeld et al., 2017; Iliff et al., 2012; Wilcock and Griffin, 2013). In contrast, in the occipital cortex, the AC group showed a greater proportion of ‘NEF Associated’ AQP4, indicating potential regional differences in astrocytic function even in the absence of overt pathology.

In the DS group, a predominance of ‘NEF Associated’ AQP4 was observed in both regions, with a higher proportion in the frontal cortex compared to the occipital cortex. This pattern was more pronounced in the frontal cortex relative to the LOAD group, suggesting that astrocytic dysfunction may occur earlier or be more pronounced in DS (Zeppenfeld et al., 2017). Semi-quantitative analysis confirmed that ‘NEF Associated’ AQP4 distribution was higher in the occipital cortex of DSAD and LOAD cases compared to DS cases. In contrast, ‘Endfoot Associated’ AQP4 distribution remained more frequent in the frontal cortex than in the occipital cortex for these groups. These findings highlight regional and diagnostic group-specific differences in AQP4 distribution patterns, with greater ‘NEF Associated’ AQP4 observed in DSAD and LOAD cases in the occipital cortex. These observations suggest AD pathology amplifies astrocytic changes and alters regional AQP4 distribution patterns, potentially contributing to impaired glymphatic function (Jessen et al., 2015; Smith et al., 2019).

The observed patterns of AQP4 distribution in DS and DSAD, particularly the increased presence in astrocytic soma and proximal branches compared to endfeet, may reflect underlying astrocytic dysfunction with important implications for glymphatic clearance. While this study could not directly assess functional outcomes, these changes suggest a potential link between altered AQP4 distribution and Aβ pathology.

This altered distribution may also be driven by chronic inflammation or genetic factors unique to DS. Chromosome 21 harbors several genes implicated in astrocytic function (e.g., S100β) and inflammatory pathways, such as Interleukin-10 receptor beta (IL-10Rβ), which could contribute to the early astrocytic changes observed in DS (Wilcock and Griffin, 2013; Griffin et al., 1989). Such alterations could differentiate the astrocytic response in DS from that seen in LOAD, impairing glymphatic clearance and exacerbating Aβ accumulation.

### 4.5. Study summary and implications

In conclusion, our study demonstrates that higher AQP4 protein levels with aging, along with shifts in AQP4 distribution in AD, may reflect disrupted astrocytic function. Higher AQP4 levels occur earlier with age and are more pronounced in DS and DSAD. AQP4 may contribute to the relationship between PVS widening observed by MRI and the accumulation of Aβ; however, direct mechanistic links remain to be established. These findings align with previous studies reporting that astrocytic proteins involved in the regulation of the extracellular environment, such as AQP4, are increased in the middle temporal gyrus of individuals with AD (Liu et al., 2025). These studies underscore the critical role of astrocytic proteins in maintaining extracellular homeostasis and suggest that disruptions to these processes may contribute to AD pathology (Liu et al., 2025). Similarly, our findings suggest that AQP4 distribution in DS and DSAD may exacerbate glymphatic dysfunction, impair Aβ clearance, and contribute to early-onset AD pathology.

The interplay among AQP4 dysfunction, PVS widening, and impaired Aβ clearance highlights the importance of investigating astrocytic changes as central mechanisms in AD pathology progression, particularly in DS. These findings underscore the need for more inclusive research addressing the unique biological and clinical needs of individuals with DS to ensure that advancements in understanding and treating AD are equitably applied to all affected populations.

### 4.6. Limitations

This study provides important insights into AQP4 distribution in DS and AD, though some limitations remain. The use of human post-mortem brain tissue restricts the ability to assess the functional implications of altered AQP4 protein levels and distribution. Clinical metadata, including cause of death and comorbidities, were variably available across brain banks and therefore were not included as covariates. During case selection, particularly from NIH NeuroBioBank, cases with neurological-related causes of death were excluded. Incomplete coverage of agonal state and comorbidity data, which may influence astrocyte responses, is an inherent limitation of human post-mortem studies.

PMI is an additional factor that must be considered in post-mortem studies. Although our analyses showed that group-level differences in AQP4 and Aβ expression remained significant after controlling for PMI, correlations with PMI highlight the potential for degradation-related bias. These associations were largely driven by younger cases from a single source with longer PMIs, rather than reflecting uniform degradation across the cohort. This limitation is inherent to human post-mortem research and underscores the importance of complementary model systems to validate these findings.

In addition, while we examined correlations between overall AQP4 expression and Aβ burden, analyses of AQP4 distribution (endfoot vs. non-endfoot) relative to Aβ levels were limited by the small subset of cases with matched data. Future work with larger, parallel-stained cohorts will be necessary to fully address this question.

Finally, while our analyses were based on validated imaging approaches, complementary quantification methods such as qPCR or Western blot could provide additional confirmation in future work.

While our data support distribution shifts across diagnostic groups, future work using live-cell models and mechanistic assays will be essential to evaluate causal relationships and therapeutic potential.

## Supplementary Material

1

## Figures and Tables

**Table 1:** Cohort Demographic and Pathological Features.

Frontal Cortex	Age Range (years)	No.	Age, Median (IQR)*	Sex Balance (Male/ Female)	PMI, Median (IQR)
**Occipital Cortex**	Young Control (< 39)	17	22.0 (11.0–28.5)	(8/9)	14.00 (9.0–21.0)
	Middle-Aged Control (40–60)	19	51.0 (42.0–64.0)	(11/8)	15.00 (6.6–20.0)
	Aged Control (70–92)	18	84.0 (77.5–88.3)	(8/10)	3.69 (2.44–5.33)
	Down Syndrome (< 39)	16	21.9 (2.0–37.5)	(10/6)	15.50 (12.3–24.0)
	Down Syndrome with AD (42–70)	17	53.0 (49.0–56.0)	(9/8)	4.65 (3.9–11.0)
	Late-Onset Alzheimer’s Disease (65–96)	16	81.0 (77.0–86.0)	(13/6)	5.58 (4.0–8.0)
	Young Control (< 39)	18	22.0 (5.5–24.5)	(12/6)	20.0 (12.0–24.0)
	Middle-Aged Control (40–60)	18	53.9 (44.6–64.2)	(11/7)	15.0 (5.8–20.0)
	Aged Control (70–92)	16	81.5 (76.8–89.0)	(5/11)	4.5 (3.0–5.1)
	Down Syndrome (< 39)	11	19.87 (9.0–25.0)	(9/2)	24.0 (14.0–28.0)
	Down Syndrome with AD (42–70)	17	53.0 (49.0–70.0)	(7/10)	7.0 (4.2–18.9)
	Late-Onset Alzheimer’s Disease (65–96)	14	79.0 (75.8–82.3)	(10/4)	5.1 (3.9–8.4)

* IQR indicates Interquartile Range.

## Data Availability

Data will be made available on request.

## Appendix A. Supplementary data

Supplementary data to this article can be found online at https://doi.org/10.1016/j.nbd.2025.107114.
