# Supplementary material for: Regional differences in astrocytic Aquaporin-4 protein levels and distribution in aging and Alzheimer’s disease in down syndrome
Source: Neurobiol Dis. Author manuscript; Available in PMC 2026 Jul 7. (PMC13341133; doi:10.1016/j.nbd.2025.107114)
Supplement: 1 [file NIHMS2183332-supplement-1.pdf]

Supplement Figure 1.

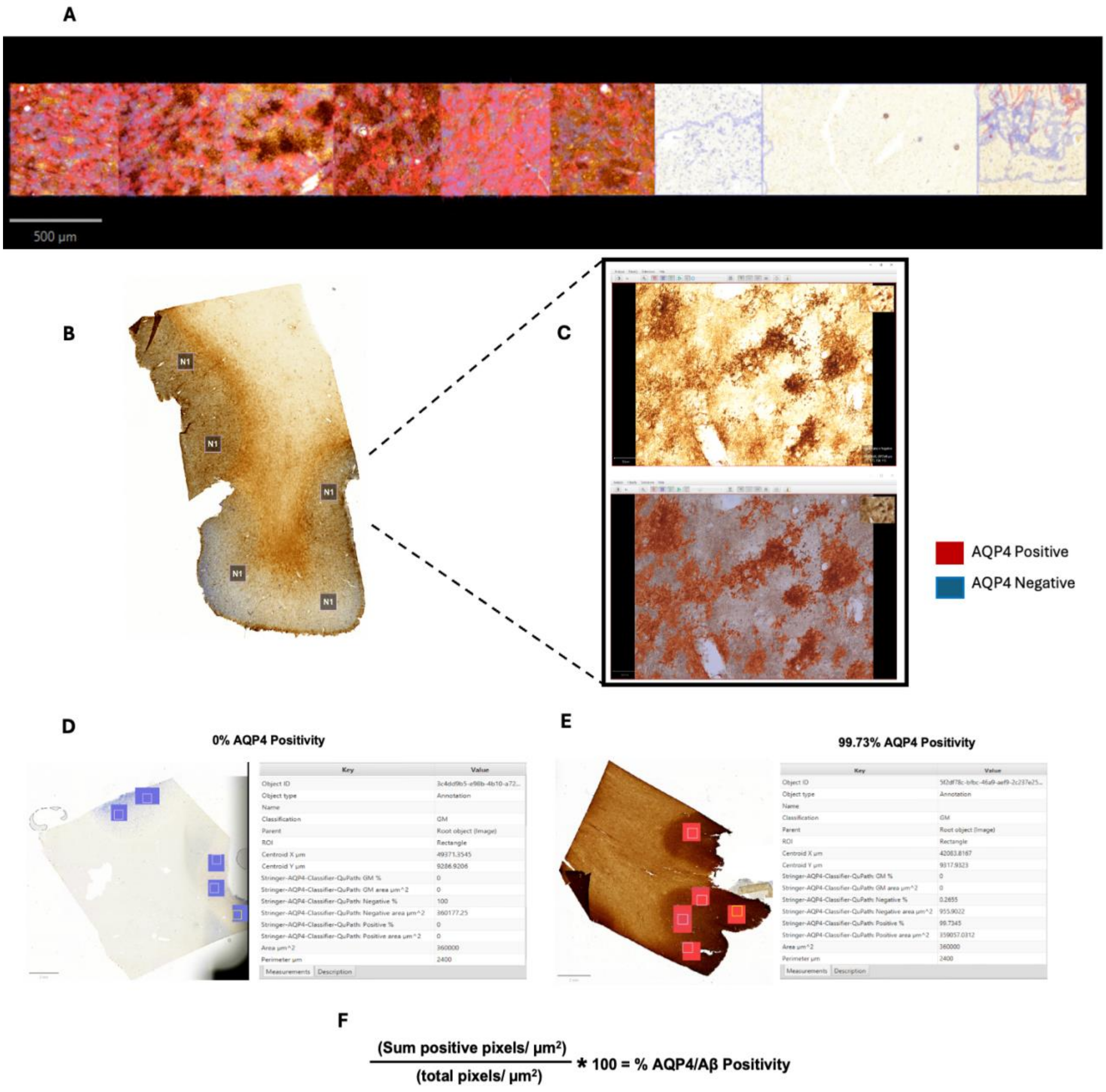

Supplement Figure 1. Training and Validation of AI Classifiers for AQP4 and Aβ Using QuPath. **(A)** Ten whole-slide images of tissue with varying protein expression levels of AQP4 and Aβ were used to train separate pixel classifiers in QuPath Version 3.0. The classifiers were trained to recognize AQP4 and Aβ, respectively, by manually tracing positive (red) and negative (blue) regions across the ten whole-slide images as demonstrated with AQP4 in this figure. **(B)** A blinded observer placed five 600μm^2 boxes randomly within the gray matter from both frontal and occipital cortical tissue across diagnostic groups. **(C)** The trained classifier was applied to each box to identify both AQP4-positive and -negative pixels per μm^2 for each box. **(D–E)** Representative classifier output for (D) a case with 0% AQP4 positivity and (E) a case with 99.73% AQP4 positivity, including classifier overlays and corresponding annotation data showing percent positivity. **(F)** AQP4 and Aβ percent positivity was calculated as the percentage of positive pixels relative to total pixels within each box. **(A-F)** All images in this figure represent AQP4 classifier training and output.

Supplement Figure 2.

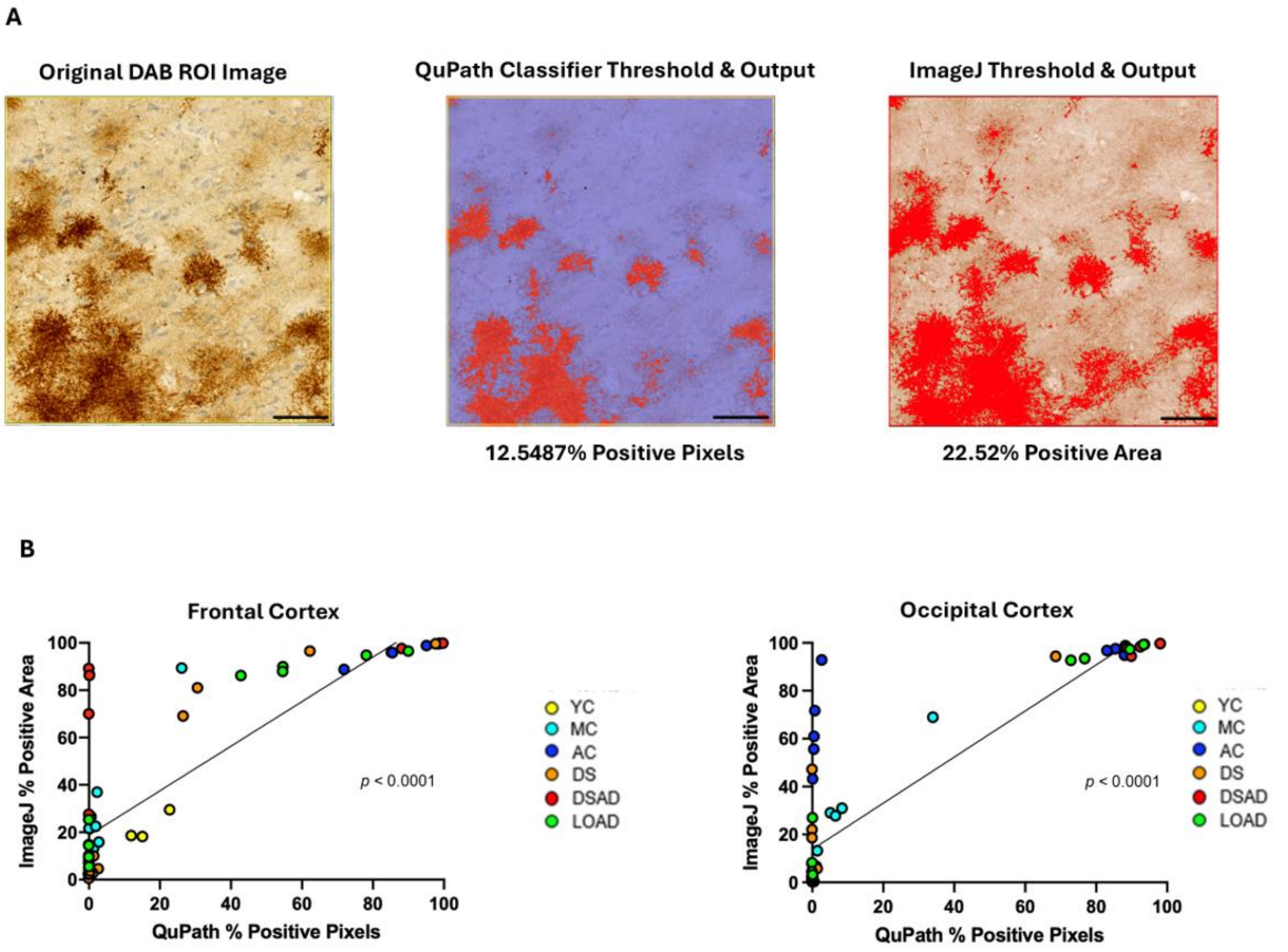

Supplement Figure 2. Validation of AQP4 QuPath Quantification Using ImageJ Thresholding as an Independent Analysis Approach. (A) Representative ROI from the main analysis showing how AQP4 labeling was quantified using two independent approaches. Left: Original DAB-stained ROI. Middle: QuPath classifier overlay (% positive pixels: 12.55%). Right: ImageJ threshold-based overlay (% positive area: 22.52%). Both methods identified overlapping astrocytic regions. Scale bars at 100 µm. **(B)** Correlation between QuPath and ImageJ quantification of AQP4 positivity across 60 ROIs from the frontal and occipital cortex. QuPath values represent % positive pixels from a trained classifier; ImageJ values represent % positive area using fixed thresholding. A strong linear correlation was observed in the frontal cortex (n=60 ROIs, $r = 0.89$, $R^2 = 0.79$, 95% CI 0.73 to 0.90, $p < 0.0001$) and occipital cortex (n=60 ROIs, $r = 0.89$, $R^2 = 0.69$, 95% CI 0.79 to 0.90, $p < 0.0001$). Linear regression analysis revealed a slope of 0.94 and intercept of 18.56 for the frontal cortex ($R^2 = 0.69$), and a slope of 0.96 and intercept of 13.61 for the occipital cortex ($R^2 = 0.79$). This indicates ImageJ values were systematically higher but proportionally related. These results validate agreement between the quantification methods. Statistical analyses were performed using Pearson correlation and simple linear regression.

Supplement Figure 3.

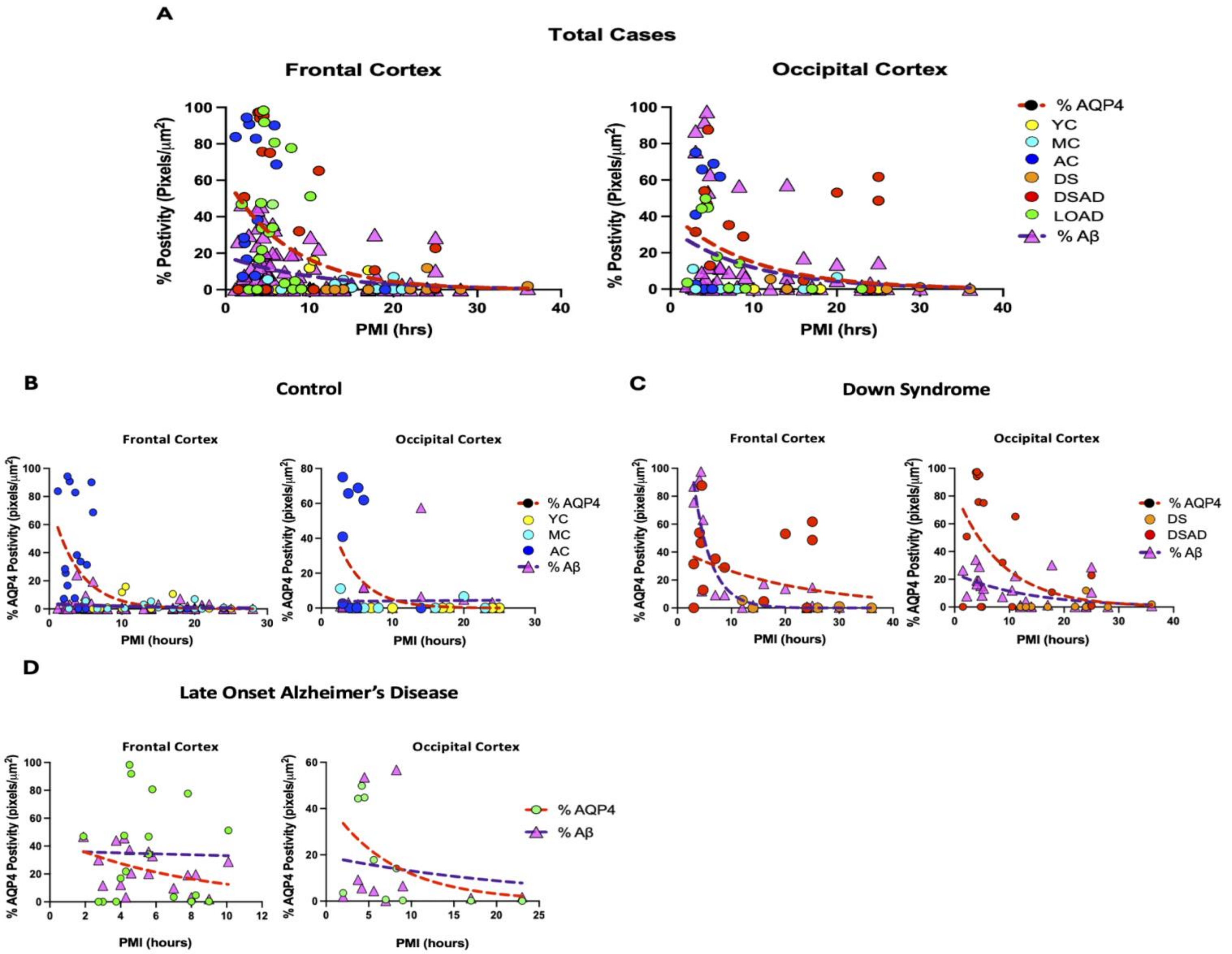

Supplement Figure 3. PMI is negatively correlated with AQP4 and Aβ protein levels. (A) Color-coded spheres represent each diagnostic group for AQP4, and pink triangles represent each diagnostic group for Aβ. AQP4 and Aβ protein levels across all groups were significantly and negatively correlated with PMI in both the frontal (%AQP4: r = - 0.44, R^2 = 0.24, p < 0.001; %Aβ: r = - 0.33, R^2 = .14, p < .001) and occipital cortices (%AQP4: r = - 0.35, R^2 = 0.15, p = 0.007; %Aβ: r = - 0.32, R^2 = 0.11, p = 0.02). **(B)** AQP4 protein levels were significantly and negatively correlated with PMI in the control group in both frontal (%AQP4: r = - 0.53, R^2 = 0.33, p < 0.001) and occipital cortices (%AQP4: r = - 0.51, R^2 = 0.24, p = 0.007). Aβ protein levels were also negatively but not significantly correlated with PMI in both the frontal (%Aβ: r = - 0.024, R^2 = 0.04, p = 0.87) or occipital cortices (%Aβ: r = - 0.04, R^2 = 0.37, p = 0.85). **(C)** AQP4 and Aβ protein levels were significantly and negatively correlated with PMI in the DS group for frontal (%AQP4: r = - 0.40, R^2 = 0.37, p = 0.03; %Aβ: r = - 0.54, R^2 = 0.26, p = 0.003) and occipital cortices (%AQP4: r = - 0.28, R^2 = 0.13, p = 0.23; %Aβ: r = - 0.44, R^2 = 0.19, p = 0.07). **(D)** AQP4 protein levels was significantly PMI in the LOAD group in the frontal cortex (%AQP4: r = 0.20, R^2 = 0.001, p = 0.07). AQP4 protein levels were significantly and negatively correlated with PMI in the occipital cortex (%AQP4: r = - 0.76, R^2 = 0.30, p = 0.015) and Aβ protein levels were negatively but not significantly correlated with PMI in the occipital cortex (%Aβ: r = - 0.30, R^2 = 0.032, p = 0.43). Data were analyzed via non-linear regression and Spearman's rho.

Supplement Figure 4.

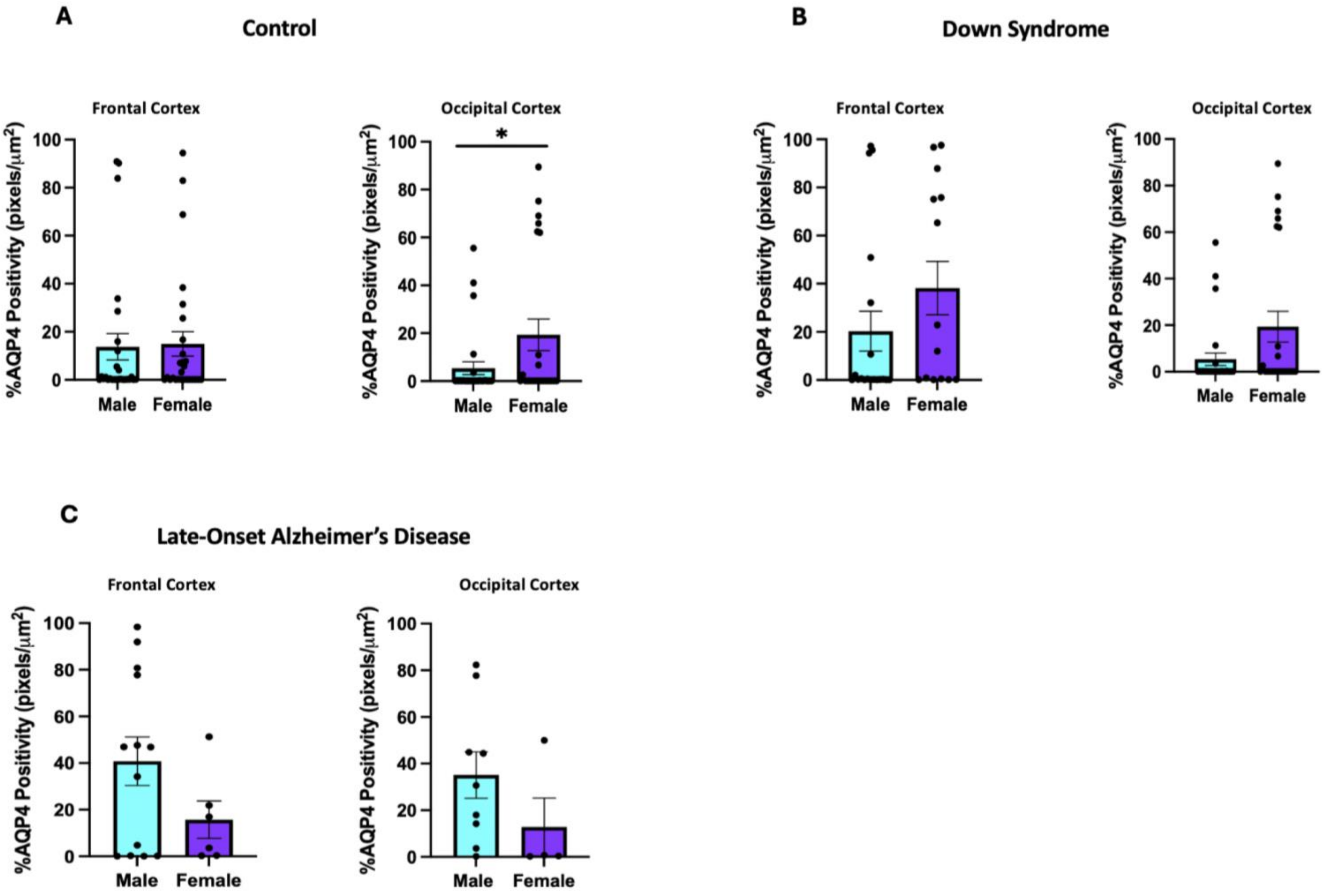

Supplement Figure 4. Sex differences in AQP4 protein levels are present in the control group in the occipital cortex. (A) AQP4 protein levels in the control group frontal cortex did not significantly differ by sex (male n = 26, female n = 26, $U = 320.5$, $p = 0.75$), but was significantly higher in females than males in the occipital cortex (male n = 27, female n = 27, $U = 237.5$, $p = 0.017$). **(B)** AQP4 protein levels did not significantly differ by sex in the DS group for either frontal (male n = 19, female n = 14, $U = 94$, $p = 0.16$) or occipital cortices (male n = 16, female n = 12, $U = 64$, $p = 0.15$). **(C)** AQP4 protein levels did not significantly differ by sex in the LOAD group for either frontal (male n = 13, female n = 6, $U = 30$, $p = 0.47$) or occipital cortices (male n = 9, female n = 4, $U = 10$, $p = 0.26$). Sex differences in AQP4 protein levels were assessed via the two-tailed Mann-Whitney U test.

Supplement Table 1. Frontal Cortex AQP4 Distribution

Group	Association	Cases (n=)	Avg Age (years)	Sex	Avg PMI (hrs)
**Aged Control**					
	No AQP4	0	-	-	-
	Endfoot	10	82.5	4 Male/6 Female	3.2
	NEF	8	82.1	4 Male/4 Female	7
**Down Syndrome**					
	No AQP4	4	16.5	3 Male/1 Female	20
	Endfoot	2	26	0 Male/2 Female	13.4
	NEF	12	27.5	7 Male/5 Female	17.5
**Down Syndrome with AD**					
	No AQP4	0	-	-	-
	Endfoot	5	52.4	3 Male/2 Female	5.7
	NEF	14	52.8	6 Male/8 Female	10.5
**Late-Onset AD**					
	No AQP4	1	83	1 Male/0 Female	9
	Endfoot	7	80.6	5 Male/2 Female	4.6
	NEF	11	79.1	8 Male/3 Female	7.5

Supplement Table 2. Occipital Cortical AQP4 Distribution

Group	Association	Cases (n=)	Avg Age (years)	Sex	Avg PMI (hrs)
**Aged Control**					
	No AQP4	0	-	-	-
	Endfoot	6	82	1 Male/5 Female	3.3
	NEF	11	83.3	5 Male/6 Female	8
**Down Syndrome**					
	No AQP4	5	9.4	4 Male/1 Female	19.8
	Endfoot	3	33.3	0 Male/3 Female	16.4
	NEF	6	27.6	5 Male/1 Female	22
**Down Syndrome with AD**					
	No AQP4	0	-	-	-
	Endfoot	3	50.3	1 Male/2 Female	10.3
	NEF	17	54.4	9 Male/8 Female	10
**Late-Onset AD**					
	No AQP4	2	80.5	1 Male/1 Female	9
	Endfoot	2	83.5	2 Male/0 Female	3.1
	NEF	12	76.9	8 Male/4 Female	9.7
